# Association between localised retinal nerve fibre layer defects and cardiovascular risk factors

**DOI:** 10.1038/s41598-019-55846-9

**Published:** 2019-12-18

**Authors:** Joo Youn Shin, Jonghyun Lee, Chan Joo Lee, Sungha Park, Suk Ho Byeon

**Affiliations:** 1 Department of Ophthalmology, Ilsan Paik Hospital, Inje University College of Medicine, Goyang, South Korea; 2 Department of Ophthalmology, The Institute of Vision Research, Yonsei University College of Medicine, Seoul, South Korea; 3 Department of Health Promotion, Severance Hospital, Seoul, Republic of Korea; 4 Cardiology Division, Severance Cardiovascular Hospital and Cardiovascular Research Institute, Yonsei University College of Medicine, Seoul, Republic of Korea

**Keywords:** Optic nerve diseases, Eye manifestations

## Abstract

Localised retinal nerve fibre layer defects (RNFLDs) are reported to indicate the degree of glaucomatous damage but can also be sequelae of retinal vascular insufficiency as a result of systemic vascular factors. We investigated the association between RNFLDs and cardiovascular risk factors. RNFLDs were detected in 440 (29.2%) of 1508 subjects. The presence of RNFLDs was associated with higher HbA_1c_ (odds ratio [OR] 1.289, *p* < 0.001), higher 24-h mean systolic blood pressure (SBP; OR 1.013, *p* < 0.005), and lower estimated glomerular filtration rate (eGFR; OR 0.995, *p* < 0.005). An increasing number of RNFLDs was correlated with higher SBP (r = 0.186, *p* < 0.001), higher HbA_1c_ (r = 0.128, *p* < 0.010), lower eGFR (r = −0.112, *p* < 0.020), and younger age (r = −0.303, *p* < 0.001). Subjects with RNFLDs had a higher predicted 10-year risk of atherosclerotic cardiovascular disease than did those without RNFLDs (9.7% vs 7.9%, *p* = 0.008 in middle-aged subjects, 25.6% vs 23.2%, *p* = 0.040 in older subjects). In conclusion, RNFLDs are associated with SBP, eGFR, and HbA_1c_. Concomitant cardiovascular risk factors should be considered when evaluating patients with localised RNFLDs.

## Introduction

Localised retinal nerve fibre layer defects (RNFLDs) have been reported to be a sensitive indicator of the degree of glaucomatous damage in patients with glaucomatous optic neuropathy^1^. However, these defects are not pathognomonic of glaucoma and can also be sequelae of retinal vascular insufficiency^2^. In an experiment in rhesus monkeys, chronic arterial hypertension and atherosclerosis were reported to lead to localised RNFLDs without glaucomatous changes in the neuroretinal rim or peripapillary atrophy^3^. Localised RNFLDs can also develop after appearance of retinal cotton-wool spots in patients with systemic hypertension or diabetes mellitus (DM)^2,4^.

Previous studies have suggested an association between localised RNFLDs and risk factors for cardiovascular disease, including high blood pressure (BP) and diabetes^5,6^. Localised RNFLDs have also been reported to be associated with cerebral small vessel disease and stroke^7,8^, suggesting that these lesions are potential biomarkers of cardiovascular risk. However, most of the studies performed to date have analysed a limited number of subjects or have not fully evaluated the various systemic factors.

The Cardiovascular and Metabolic Disease Etiology Research Center-High Risk Cohort (CMERC-HI) study is a prospective cohort investigation of the value of known and novel predictors of clinical outcomes in subjects at risk of cardiovascular disease. The aim of the present study was to evaluate the association between localised RNFLDs and various cardiovascular risk factors using the baseline CMERC-HI data to improve understanding of the pathogenesis of RNFLDs.

## Results

### Subject characteristics at baseline

Localised RNFLDs were detected in 440 (29.2%) of the 1508 subjects (Table 1). Subjects with RNFLDs had a higher glycated haemoglobin (HbA_1c_) level (*p* = 0.003), higher BP (mean 24-h systolic BP [SBP] and diastolic BP [DBP], daytime SBP, night-time SBP and DBP [*p* < 0.001], and daytime DBP [*p* = 0.007]) and a lower estimated glomerular filtration rate (eGFR; *p* < 0.001) than did those without RNFLDs. There was no difference in age (*p* = 0.131), sex (*p* = 0.424), rate of hypertension (*p* = 0.968), rate of DM (*p* = 0.102), lipid profile values, or urinary albumin to creatinine ratio between the two groups.

**Table 1:** Baseline characteristics according to the presence of localised retinal nerve fibre layer defects.

Variable	RNFLD (−)	RNFLD (+)	*p*-value
Subjects, n	1068	440	
Age, years	59.76 ± 11.55	58.76 ± 11.80	0.131
Sex, male, n (%)	594 (55)	255 (58)	0.424
BMI	25.31 ± 3.62	25.08 ± 3.79	0.234
Smoking status			
Never/Past/Current, n	580/ 349/ 139	231/150/59	0.813
Hypertension, n (%)	997 (93)	411 (93)	0.968
*24-h ambulatory BP monitoring*			
24-h SBP, mmHg	129.16 ± 14.21	133.64 ± 15.35	<0.001*
24-h DBP, mmHg	77.80 ± 8.03	79.60 ± 8.63	<0.001*
Daytime SBP, mmHg	133.84 ± 14.31	137.91 ± 15.21	<0.001*
Daytime DBP, mmHg	80.93 ± 8.44	82.30 ± 8.87	0.007*
Night-time SBP, mmHg	120.33 ± 16.43	125.83 ± 18.27	<0.001*
Night-time DBP, mmHg	71.93 ± 8.87	74.87 ± 10.01	<0.001*
*Diabetes*			
DM, n (%)	388 (36)	180 (41)	0.102
HbA_1c_, %	6.06 ± 0.84	6.25 ± 1.12	0.003*
Glucose, mg/dL	109.80 ± 26.73	114.82 ± 37.11	0.012*
*Kidney function*			
BUN, mg/dL	24.01 ± 16.25	28.98 ± 21.49	<0.001*
Creatinine, mg/dL	1.83 ± 2.46	2.55 ± 3.34	<0.001*
eGFR, mL/min/1.73 m^2^	67.35 ± 33.42	58.16 ± 35.56	<0.001*
Urinary ACR (mg/g)	309.96 ± 788.4	385.7 ± 907.5	0.127
*Lipid profile*			
Total cholesterol, mg/dL	171.69 ± 36.06	172.43 ± 35.59	0.723
HDL cholesterol, mg/dL	49.11 ± 13.56	47.55 ± 13.19	0.806
LDL cholesterol, mg/dL	94.23 ± 29.07	95.09 ± 29.87	0.622
Triglycerides, mg/dL	138.52 ± 88.12	145.05 ± 79.81	0.199

The data are shown as the mean ± standard deviation or the number (percentage). *P < 0.05. ACR, albumin to creatinine ratio; BMI, body mass index; DBP, diastolic blood pressure; DM, diabetes mellitus; eGFR, estimated glomerular filtration rate; HbA_1c_, glycated haemoglobin; HDL, high-density lipoprotein; LDL, low-density lipoprotein; RNFLD, localised retinal nerve fibre layer defect; SBP, systolic blood pressure

### Association between RNFLDs and cardiovascular risk factors

In the regression analysis, localised RNFLDs was the dependent variable and other parameters that were significantly associated with localised RNFLDs in the univariate analysis (Table 1) and were clinically significant were the independent variables. In the multivariate logistic regression, higher HbA_1c_ (odds ratio [OR] 1.289, 95% confidence interval [CI] 1.13–1.47, *p* < 0.001), higher 24-h mean SBP (OR 1.013, 95% CI 1.004–1.022, *p* = 0.005), and lower eGFR (OR 0.995, 95% CI 0.991–0.998, *p* = 0.005; Table 2) were found to be associated with the presence of localised RNFLDs.

**Table 2:** Factors associated with presence of localised retinal nerve fibre layer defects using multivariate logistic regression analysis.

	Unit change	OR	95% CI	*P*-value
Age	Per 1-year increment	0.991	0.98–1.002	0.110
Sex	Female vs male	0.950	0.74–1.23	0.695
BMI	Per 1-kg/m^2^ increment	0.974	0.94–1.01	0.144
HbA_1c_	Per 1% increment	1.289	1.13–1.47	<0.001*
Mean 24-h SBP	Per 1-mmHg increment	1.013	1.004–1.022	0.005*
eGFR	Per 1-mL/min/1.73 m^2^ increment	0.995	0.991–0.998	0.005*

*p < 0.05. BMI, body mass index; CI, confidence interval; HbA_1c_, glycated haemoglobin; OR, odds ratio; SBP, systolic blood pressure; eGFR, estimated glomerular filtration rate.

Given the association of presence of localised RNFLDs with higher HbA_1c_, we performed regression analysis by dividing the subjects into a diabetes group and a non-diabetes group. In the non-diabetes group, the presence of localised RNFLDs was associated with a higher 24-h mean SBP (OR 1.013, 95% CI 1.002–1.024, *p* = 0.024) and a lower eGFR (OR 0.994, 95% CI 0.989–0.998, *p* = 0.007) after adjustment for age, sex, and body mass index (BMI). In the diabetes group, the presence of RNFLDs was significantly associated with a higher HbA_1c_ (OR 1.446, 95% CI 1.195–1.749, *p* < 0.001) and a higher 24-h mean SBP (OR 1.014, 95% CI 1.0–−1.028, *p* = 0.045) after adjustment for age, sex, BMI, and eGFR (OR 0.994, 95% CI 0.988–1.00, *p* = 0.057, for eGFR).

### Correlation between number of RNFLDs and cardiovascular risk factors

As with the presence of RNFLDs, the number of RNFLDs was significantly correlated with higher HbA_1c_ (r = 0.128, *p* = 0.010), higher mean 24-h SBP (r = 0.186, *p* < 0.001), and lower eGFR (r = −0.112, *p* = 0.020). The increasing number of RNFLDs was also correlated with younger age (r = −0.303, *p* < 0.001). No correlation was detected between the number of RNFLDs and sex (*p* = 0.924) or BMI (*p* = 0.972; Table 3).

**Table 3:** Correlation between number of localised retinal nerve fibre layer defects and cardiovascular risk factors.

Variables	RNFLDs, n			Pearson’s coefficient	*p*-value
	1	2	≥3		
Age (years)	61.8 ± 10.5	57.8 ± 12.0	52.3 ± 11.9	−0.303	<0.001
Sex (M/F)	125/92	84/57	46/36	0.005	0.924
BMI	25.0 ± 3.3	25.2 ± 3.9	25.0 ± 4.6	0.002	0.972
Mean 24-h SBP (mmHg)	131.5 ± 14.4	133.9 ± 14.0	139.5 ± 18.7	0.186	<0.001
HbA_1c_ (%)	6.10 ± 0.85	6.36 ± 1.28	6.45 ± 1.39	0.128	0.010
eGFR (mL/min/1.73 m^2^)	61.1 ± 34.0	58.7 ± 37.3	49.7 ± 35.5	−0.112	0.020

*p < 0.05. RNFLD, retinal nerve fibre layer defect; BMI, body mass index; HbA_1c_, glycated haemoglobin; SBP, mean systolic blood pressure; eGFR, estimated glomerular filtration rate.

### Predicted 10-year risk of atherosclerotic cardiovascular disease

Six hundred and twenty-three subjects were aged 40–59 years (designated the middle-aged group) and 781 were aged 60–79 years (designated the older group). RNFLDs were observed in 31.5% of the middle-aged group and 26.9% of the older group. The mean ASCVD risk score for subjects with RNFLDs was 9.7% in the middle-aged group and 25.6% in the older group; the score was significantly higher in subjects with RNFLDs than in those without RNFLDs in both age groups (*p* = 0.008 and *p* = 0.040, respectively, Table 4).

**Table 4:** Predicted 10-year risk of ASCVD by ASCVD score according to the presence of localised retinal nerve fibre layer defects.

Age group	n	10-year risk predicted by ASCVD score (%)	*p*-value
40–59 years			
RNFLD (−)	427	7.9 ± 7.1	0.008*
RNFLD (+)	196	9.7 ± 8.2	
60–79 years			
RNFLD (-)	571	23.2 ± 14.5	0.040*
RNFLD (+)	210	25.6 ± 14.6	

*p < 0.05. RNFLD, retinal nerve fibre layer defect; ASCVD, atherosclerotic cardiovascular disease.

## Discussion

The present study investigated the association between localised RNFLDs and cardiovascular risk factors and showed that RNFLDs were associated with 24-h mean SBP, HbA_1c_, and eGFR values after adjustment. An increasing number of RNFLDs was also correlated with higher SBP, lower eGFR, higher HbA_1c,_ and younger age. The 10-year predicted risk of cardiovascular disease using the ASCVD score was significantly higher in subjects with localised RNFLDs than in those without RNFLDs. Our results suggest that concomitant cardiovascular risk factors should be considered when evaluating patients with localised RNFLDs.

Localised RNFLDs have been reported to be associated with arterial hypertension^5,9,10^. Chronic arterial hypertension and atherosclerosis led to development of localised RNFLDs in a rhesus monkey model^3^, and localised RNFLDs were also reported to be associated with increasing grades of arterial hypertension^5^. The most common causes of RNFLDs in patients with uncontrolled hypertension are cotton-wool spots^11^ and/or hypertensive optic neuropathy^12^. The authors of a study in primates with experimental renovascular hypertension suggested that autoregulatory vasoconstriction in the superficial arterioles resulting from high BP leads to occlusive sequelae and ischemic damage in the inner retina^5,13^. Our finding of an association between localised RNFLDs and higher BP, i.e., the probability of having RNFLDs increases by 1.13-fold with a 10-mmHg increment in blood pressure, is in line with earlier reports.

Analysis of our 24-h ambulatory BP monitoring results revealed that the presence of RNFLDs was associated with persistently high SBP and DBP. It has been reported that ambulatory BP provides better prognostic information than does BP recorded in the office in patients with cardiovascular disease and that high BP at night increases the cardiovascular risk in patients with high ambulatory daytime BP^14^. Therefore, the finding in our study that both daytime and night-time BP values were higher in subjects with RNFLDs than in those without RNFLDs suggests an increased likelihood of cardiovascular events, as seen in previous studies that have identified an association between RNFLDs and stroke^7,8^.

The presence of localised RNFLDs was also associated with the HbA_1c_ value. Although the mechanism of RNFLDs in subjects with diabetes is not completely understood, oxidative stress, accumulation of advanced glycation end products, and impaired retrograde axonal transport of ganglion cells have been suggested to play a role^2,15,16^. Acute nonperfusion of the retina causes a blockage of axoplasmic transport and formation of cotton-wool spots, and it has been reported that approximately 22% of cotton-wool spots cause clinically visible RNFLDs in patients with diabetic retinopathy^2^. The frequency of these changes was reported to depend on the severity and duration of disease and on the BP^17^. Increased BP has been hypothesised to damage the retinal capillary endothelial cells in patients with diabetes^18^, and our finding that the presence of RNFLDs in subjects with diabetes was also associated with SBP after adjustment for HbA_1c_ is consistent with the mechanism proposed in previous studies.

There was an independent association between the presence of RNFLDs and a lower eGFR, particularly in subjects without diabetes after adjustment for other cardiovascular risk factors. Pathogenic abnormalities, including dysfunction of the renin-angiotensin system, atherosclerosis, and oxidative stress, may occur throughout the body; microvascular damage from hypertension, DM, and other processes affecting the retinal and renal vasculature may be useful indicators of cumulative damage. Previous studies have shown that the RNFL is thinner in patients who have chronic renal failure without DM^19^ and in those receiving haemodialysis^20^ than in the normal population. The authors of those studies suggested subclinical ischemic retinopathy as the reason for the reduction in retinal thickness in patients with chronic kidney disease. Our result is consistent with their findings and suggests that localised RNFLDs could indicate generalised systemic microvascular injury and also reflect underlying renal injury^21^.

As with the presence of RNFLDs, the number of RNFLDs was significantly correlated with cardiovascular risk factors including higher SBP, lower eGFR, and higher HbA1_c_, supporting the notion that RNFLDs could be a potential biomarker of cardiovascular risk^22^. On the other hand, the number of RNFLDs was inversely correlated with age. The reason for this result is unclear, but we can speculate about the reason on the basis of the finding in a previous study that younger age was a strong independent risk factor for development of grade III/IV hypertensive retinopathy^23^. Alternatively, this finding could reflect the decreased visibility of localised RNFLDs in older individuals^8^.

Subjects with RNFLDs had a significantly higher predicted 10-year risk for ASCVD than did those without RNFLDs regardless of whether they were middle-aged or older. This result suggests that RNFLDs may have a predictive value with regard to cardiovascular risk. In particular, given our findings that the number of RNFLDs was inversely correlated with age and that the ASCVD risk score in the RNFLD group was more significant in the middle-aged group than in the older group, RNFLDs may be of more benefit for prediction of cardiovascular risk in younger, middle-aged individuals with risk factors. A longitudinal study will be needed to answer this question.

There are some limitations to our study. We included only subjects of the same ethnicity with a high risk of developing cardiovascular disease, so it may be difficult to generalise our findings. Similar studies in the general population are now needed. Furthermore, a previous study reported that the ASCVD risk score in the Korean population was overestimated by the ACC/AHA risk equations^24^, so our calculated risk score may not reflect the actual risk. We anticipate that the 5–10-year outcomes in our cohort will be needed to determine conclusively whether or not evaluation of RNFLDs would be of benefit over and above that of existing methods for prediction of cardiovascular risk. The cross-sectional study design and lack of a control group were further limitations. Moreover, although statistically significant, the odds ratios were small. However, despite these limitations, we analysed numerous cardiovascular risk factors and demonstrated associations of these factors with RNFLDs that could increase understanding of the pathogenesis of RNFLDs. The association of RNFLDs and cardiovascular risk factors should be considered when evaluating patients with RNFLDs. This association might have an impact on studies in glaucoma that use RNFLD as a diagnostic criterion; however, investigation of this was beyond the scope of our study because we excluded subjects with significant glaucomatous changes at the optic disc. We believe that future studies should investigate this possibility.

In conclusion, our findings indicate that localised RNFLDs are associated with certain cardiovascular risk factors, including SBP, eGFR, and HbA_1c_, suggesting that concomitant cardiovascular risk factors should be considered when evaluating a patient with localised RNFLDs.

## Methods

### Study populations

The Division of Cardiology, Severance Cardiovascular Hospital, Yonsei University College of Medicine, has been enrolling subjects in the CMERC-HI cohort (ClinicalTrials.gov identifier NCT02003781) since December 2013. The study protocol will be reported in detail elsewhere. This study was performed in accordance with the tenets of the Declaration of Helsinki. The Institutional Review Board at Yonsei University approved the protocol for the present study (4-2014-000) and all participants provided written informed consent.

Subjects are deemed to have cardiovascular risk factors and enrolled in the CMERC-HI cohort study if they have any of the following: high-risk hypertension (eGFR >60 mL/min/1.73 m^2^ with damage to at least one target organ or an eGFR ≤60 mL/min/1.73 m^2^); DM with albuminuria; anuric end-stage renal disease requiring dialysis; a relative who sustained an acute myocardial infarction when aged younger than 55 years (if male) or 65 years (if female); asymptomatic atherosclerotic disease (abdominal aorta diameter ≥3 cm or ankle-brachial index >0.9, carotid plaque or carotid intima-media thickness ≥0.9 mm, an asymptomatic old cerebrovascular accident, or >30% stenosis in at least one major coronary artery); rheumatoid arthritis, age >40 years, and taking methotrexate and steroids; atrial fibrillation with a CHA2DS-VASc score ≥1; and a history of kidney transplantation more than 3 months beforehand. Subjects aged >20 years who met at least one inclusion criterion were enrolled in our present study. We excluded subjects with any of the following: acute myocardial infarction, acute coronary syndrome, or symptomatic coronary artery disease, or a history of any of these diseases; symptomatic peripheral artery disease or heart failure, or a history of either of these diseases; expected survival of less than 6 months for a non-cardiovascular reason (e.g., cancer or sepsis); history of contrast allergy or side effects related to contrast materials; and current pregnancy or lactation.

In this study, we analysed the baseline data for 1508 subjects enrolled in the CMERC-HI cohort between November 2013 and June 2016 who agreed to an examination of the fundus.

### Assessment of cardiovascular risk factors

Subjects underwent a standardised interview that included questions about a known diagnosis of hypertension, DM, and chronic kidney disease, as well as the current treatment of these diseases. Anthropometric parameters, including body height and weight, were measured. The HbA_1c_ level and blood glucose, lipid, blood urea nitrogen, creatinine, and urinary albumin concentrations were measured in blood samples taken after fasting overnight and spot urine specimens. SBP and DBP were measured at 30-min intervals while subjects performed their normal daily activities, including sleep, using a 24-h ambulatory BP monitor (TM-2430, A & D Medical, Tokyo, Japan).

### Predicted 10-year risk for atherosclerotic cardiovascular disease

ASCVD is defined as a nonfatal myocardial infarction (heart attack), death from coronary heart disease, or stroke. The American College of Cardiology/American Heart Association (ACC/AHA) 2013 Pooled Cohort Equations formula estimates the 10-year risk of ASCVD in patients who have never experienced one of these events in the past based on the covariates of age, treated or untreated systolic BP level, total cholesterol and high-density lipoprotein cholesterol levels, current smoking status (yes/no), and history of diabetes (yes/no)^25^. Using this formula, the ASCVD risk score was calculated and compared between subjects with and without RNFLDs. One hundred and four of 1508 subjects were excluded from the analysis for being aged younger than 40 years or older than 80 years (ASCVD risk score not applicable; n = 94) or having a total cholesterol level <100 despite being within the appropriate age range (n = 10).

### Retinal imaging with fundus photography and spectral-domain OCT

All subjects underwent fundus photography and spectral-domain optical coherence tomography (OCT) on the same day. For both examinations, the pupils were dilated using tropicamide 1%. Two fundus photographs of each eye, the first centred on the optic disc and the second centred on the fovea, were acquired using a 45° fundus camera (Visucam^NM/FA^, Carl Zeiss Meditec, Jena, Germany). Spectral-domain OCT (Spectralis HRA HROCT; Heidelberg Engineering GmbH, Dossenheim, Germany) was used to obtain circular scans (3.4 mm in diameter) centred at the optic disc and horizontal, vertical-B, and volume scans of the macular area. The volume scans contained 49 sections (with spacing of 120 μm) of a 20 × 20° area of the macula. Bilateral fundus photographs and OCT images were available for 1503 of the 1508 subjects (5 subjects underwent examination of the fundus of only one eye because of media opacity).

**Figure 1:**
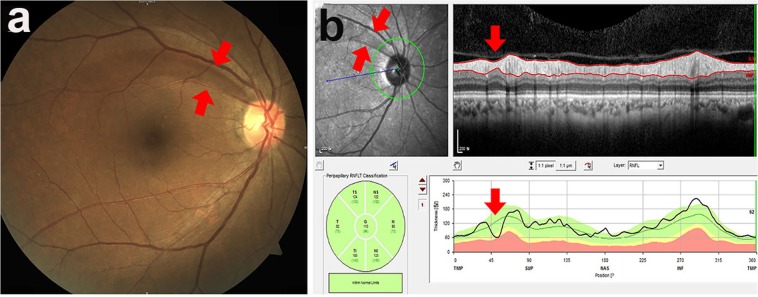
Localised retinal nerve fibre layer defect assessed using fundus photography (**a**) and spectral-domain optical coherence tomography (**b**). A localised retinal nerve fibre layer defect was defined as a dark wedge-shaped area with a connecting optic disc border on fundus photography that had a width or length of 5°–45° (**a**), which was confirmed on optical coherence tomography as an abruptly dropped contour line on the thickness profile of the retinal nerve fibre layer (**b**).

A localised RNFLD was defined as a dark wedge-shaped area with a connecting optic disc border on fundus photography that had a width or length of 5°–45°^9,26^, which was confirmed on OCT as an abruptly dropped contour line on the RNFL thickness profile (Fig. 1). The images of the fundus were assessed by an ophthalmologist (JYS) who was blinded to all subject data except for the fundus photographs and OCT images. To determine the inter-observer variability, photographs of 100 randomly selected subjects were reviewed by two blinded ophthalmologists (JYS, SHB) working independently of each other. The coefficient for inter-observer variability in detection of localised RNFLDs was 0.99. RNFLDs associated with significant glaucomatous changes at the optic disc, chorioretinal scarring, a history of uveitis, or retinal artery or vein occlusion were not regarded as localised RNFLDs in the analysis.

### Statistical analysis

The independent *t*-test was used for continuous variables and the chi-squared test for categorical variables when comparing the baseline characteristics of the subject groups with and without RNFLs. Multivariate logistic regression was used to determine the association between presence of RNFLDs and cardiovascular risk factors. ORs and 95% CIs were calculated. In multivariate logistic regression, we used the presence of RNFLDs as a dependent variable. Parameters that were significantly associated with RNFLDs in the univariate analysis and were clinically significant were used as independent variables. Therefore, subjects with higher values for one or more independent variables (e.g., systolic pressure) had higher odds of having RNFLDs. In subjects with RNFLDs, the number of RNFLDs was regarded as the number of RNFLDs in the eyes with more RNFLDs. The Pearson correlation test was performed to determine the correlation between the number of RNFLDs and cardiovascular risk factors. The ASCVD risk score was compared between the groups with and without RNFLDs using the independent *t*-test. Given that age had a strong influence on the ASCVD risk score, we divided the subjects into groups aged 40–59 years (the middle-aged group) and those aged 60–79 years (older group). The statistical analysis was performed using SPSS for Windows (version 21.0; IBM Corp., Armonk, NY). A *p-*value al0.05 was considered statistically significant.

## Data Availability

All data generated or analysed during this study are included in this article.
